# Electrocardiographic Changes after Endovascular Mechanical Thrombectomy in a Patient with Pulmonary Embolism—A Case Report and Literature Review

**DOI:** 10.3390/jcm13092548

**Published:** 2024-04-26

**Authors:** Lukas Ley, Florian Messmer, Lukas Vaisnora, Hossein Ardeschir Ghofrani, Dirk Bandorski, Michael Kostrzewa

**Affiliations:** 1 Department of Radiology, Baden Cantonal Hospital, 5404 Baden, Switzerland; florian.messmer@ksb.ch (F.M.); michael.kostrzewa@ksb.ch (M.K.); 2 Campus Kerckhoff, Justus-Liebig-University Giessen, 61231 Bad Nauheim, Germany; 3 Department of Cardiology, Baden Cantonal Hospital, 5404 Baden, Switzerland; lukas.vaisnora@ksb.ch; 4 Universities of Giessen and Marburg Lung Center (UGMLC), 35392 Giessen, Germany; h.ghofrani@kerckhoff-klinik.de; 5 Faculty of Medicine, Semmelweis University Campus Hamburg, 20099 Hamburg, Germany; dirk.bandorski@t-online.de

**Keywords:** electrocardiogram, mechanical thrombectomy, pulmonary embolism, case report

## Abstract

**Background:** Pulmonary embolism (PE) is a common disease with an annual incidence of about 1/1000 persons. About every sixth patient dies within the first 30 days after diagnosis. The electrocardiogram (ECG) is one of the first diagnostic tests performed, and is able to confirm the suspicion of PE with typical electrocardiographic signs. Some ECG signs and their regression are also prognostically relevant. Endovascular mechanical thrombectomy is one option for PE treatment, and aims to relieve right heart strain immediately. The first studies on endovascular mechanical thrombectomy using a dedicated device (FlowTriever System, Inari Medical, Irvine, CA, USA) yielded promising results. **Methods:** In the following, we report the case of a 66-year-old male patient who presented with New York Heart Association III dyspnea in our emergency department. Among typical clinical and laboratory results, he displayed very impressive electrocardiographic and radiological findings at the time of PE diagnosis. **Results:** After endovascular mechanical thrombectomy, the patient’s complaints and pulmonary hemodynamics improved remarkably. In contrast, the ECG worsened paradoxically 18 h after intervention. Nevertheless, control echocardiography 4 days after the intervention no longer showed any signs of right heart strain, and dyspnea had disappeared completely. At a 4-month follow-up visit, the patient presented as completely symptom-free with a high quality of life. His ECG and echocardiography were normal and excluded recurrent right heart strain. **Conclusions:** Overall, the patient benefitted remarkably from endovascular mechanical thrombectomy, resulting in an almost complete resolution of electrocardiographic PE signs at the 4-month follow-up after exhibiting multiple typical electrocardiographic PE signs at time of diagnosis and initial electrocardiographic worsening 18 h post successful intervention.

## 1. Introduction

Pulmonary embolism (PE) occurs with an annual incidence of 0.4–1.2/1000 persons [1]. Together with deep vein thrombosis (DVT), it is the third most common acute cardiovascular syndrome worldwide after myocardial infarction and stroke [2]. An up-to-date registry reports an overall 16% 30-day mortality rate [3]. In the European Union, PE and DVT account for up to EUR 8.5 billion in healthcare costs every year, and will probably place an even greater burden on healthcare systems in the future [1,4]. Because of its ubiquitous availability, inexpensiveness, ease of use and good diagnostic performance, the electrocardiogram (ECG) is one of the first diagnostic tests to be performed in an emergency setting, especially in patients with cardiac or respiratory symptoms. The ECG is able to confirm the suspicion of PE through typical electrocardiographic changes [1,5,6,7]. However, electrocardiographic PE signs are absent in about 15–40% of PE patients, particularly in milder courses [5,6,8,9,10]. Nevertheless, some ECG changes (e.g., sinus tachycardia, SIQIII type, right bundle branch block and T wave inversions) have prognostic significance [5,8,9,11,12,13]. A dreaded complication of PE is right heart failure, which can lead to subsequent death [1]. Endovascular mechanical thrombectomy using a dedicated device (FlowTriever System, Inari Medical, Irvine, CA, USA) is a minimally invasive treatment option for PE. Mechanical thrombectomy can result in the immediate relief of right heart strain, and early studies demonstrated high efficiency and low mortality [1,14,15,16,17]. In the following report, we present the case of a patient who displayed impressive electrocardiographic and radiological findings at the time of PE diagnosis, and benefitted remarkably from endovascular mechanical thrombectomy. To our knowledge, this is the first report on the electrocardiographic course after successful PE treatment with endovascular mechanical thrombectomy using the FlowTriever System.

## 2. Patient

**Figure 1:**
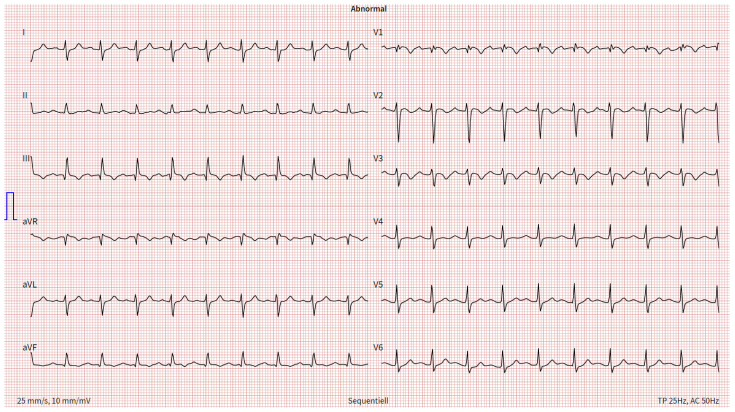
ECG at time of diagnosis. Annotation: sinus tachycardia (116 bpm); SIQIII type; no P pulmonale; P amplitude in II: 0.12 mV; incomplete right bundle branch block (QRS time: 110 ms); persistent S waves in V5/V6; negative Sokolow–Lyon index for right ventricular hypertrophy (>1.05 mV, here 0.65 mV); R V1, V2 + S I, aVL − S V1 > 0.6 mV (0.7 mV); R wave peak in V1 > 35 ms (60 ms); T wave inversions in III, aVF, V1–V3; no extrasystoles; ECG, electrocardiogram.

A 66-year-old male patient with a BMI of 27.2 kg/m^2^ presented to his family doctor with exertional dyspnea (New York Heart Association (NYHA) II) after being on a hiking vacation and was referred to our radiology department to rule out PE. However, because his dyspnea worsened significantly (NYHA III), the patient directly presented to our emergency department. No clinical evidence of acute DVT could be evaluated. However, the patient described a previous episode of sudden onset exertional dyspnea with rapid resolution 3 years ago, and previous signs and symptoms suspicious of DVT about 20 years ago. He also suffered from arterial hypertension and dyslipidemia. In the emergency room, the patient presented with sinus tachycardia (129 bpm), elevated blood pressure (149/102 mm Hg), decreased O_2_ saturation (94%) and borderline tachypnoea (20 breaths/min). Typical electrocardiographic PE signs could be found (Figure 1). 

**Figure 2:**
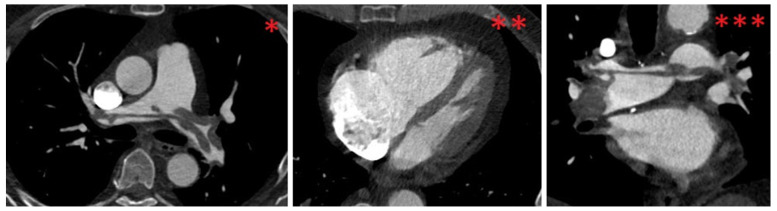
CTPA findings at time of diagnosis. * and **: axial view. ***: coronal view; bilateral, central saddle pulmonary embolism (*, ***); pulmonary artery-to-aorta diameter ratio > 1 (1.1, *); caval contrast reflux (**); D-sign (**); right ventricle-to-left ventricle diameter ratio > 1 (2.2, **); CTPA, computed tomographic pulmonary angiography.

The ECG revealed sinus tachycardia (116 bpm), an SIQIII type, an incomplete right bundle branch block (RBBB, QRS time: 110 ms) and persistent S waves in leads V5 and V6. In addition, T wave inversions (TWI) were found in leads III, aVF and V1–V3. Moreover, a parameter for right heart strain (R V1, V2 + S I, aVL − S V1) exceeded a threshold of 0.6 mV (0.7 mV), and the time to peak of the R wave in V1 was longer than a threshold of 35 ms (60 ms) [18]. Due to the suspicion of PE, Wells I and Wells II scores were assessed, and the results (6/12.5 and 3/7, respectively) indicated likely PE [19,20]. Due to the patient’s hemodynamic stability, a computed tomography pulmonary angiogram (CTPA) was performed, in which a bilateral, central saddle PE and vivid signs of right heart strain (right ventricle-to-left ventricle diameter ratio: 2.2) were observed (Figure 2). 

**Figure 3:**
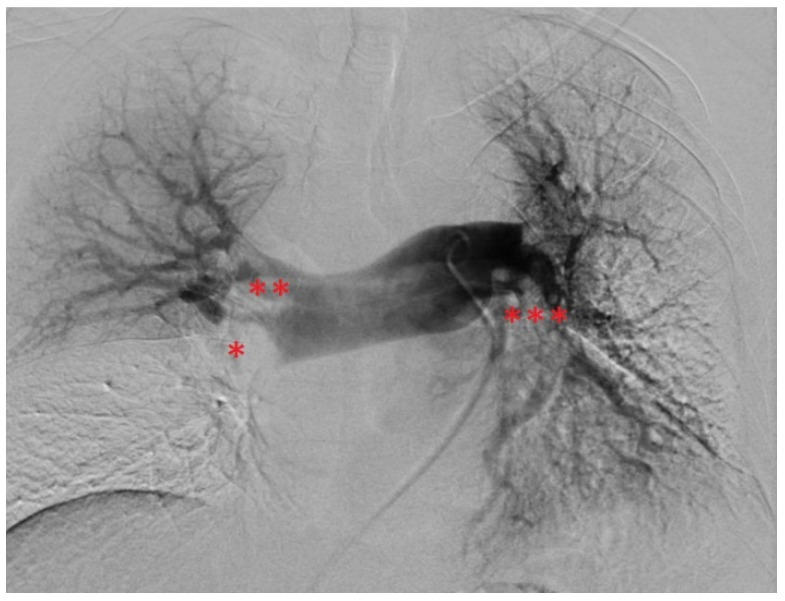
DSA findings before endovascular mechanical thrombectomy. Annotation: right interlobar artery (*) appears truncated with no parenchymal contrasting of the right lower lobe. There are significant filling defects in the right superior trunk (**), the left interlobar artery (***) and the left apical segmental arteries, also with a lack of contrasting of the corresponding pulmonary parenchyma. DSA, digital subtraction angiography.

**Figure 4:**
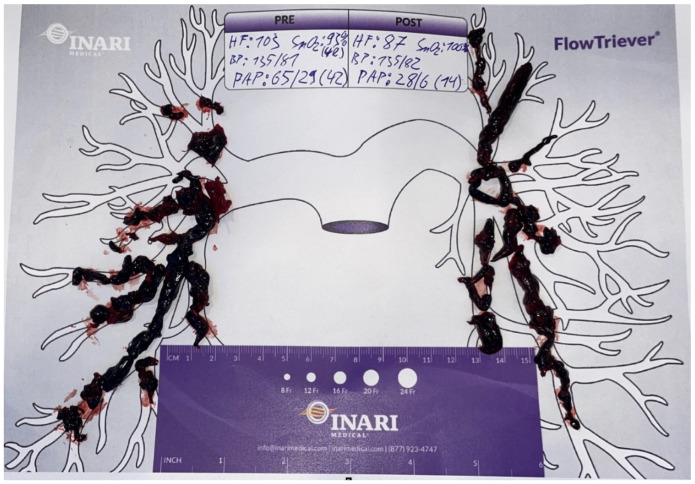
Recovered pulmonary thrombi.

**Figure 5:**
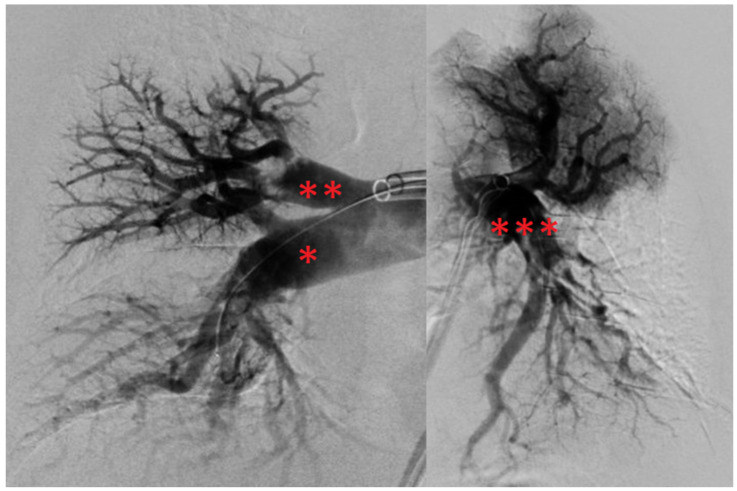
DSA findings after endovascular mechanical thrombectomy. Annotation: Near-complete contrasting of the right interlobar artery (*), the right superior trunk (**), left interlobar artery (***) and left apical segmental arteries with only small remaining filling defects corresponding to small residual thrombi. DSA, digital subtraction angiography.

Elevation of high-sensitivity cardiac troponin T (43 ng/L) and NT-pro-BNP levels (3538 ng/L), as well as the hypoxemic respiratory failure found in the arterial blood gases (pO_2_: 67 mm Hg, pCO_2_: 24 mm Hg), underpinned the diagnosis. Pulmonary Embolism Severity Index (PESI) and simplified PESI (sPESI) scores were calculated to be 96 and 1, respectively [21,22]. From his PESI score, the patient was categorized as class III, revealing a moderate 30-day mortality risk (approximately 3–7%) [1,21]. Clinically, the patient was classified as intermediate–high risk [1]. Moreover, a DVT in the medial segment of the left posterior tibial vein was detected by compression ultrasonography. Based on this, he was anticoagulated therapeutically with unfractionated heparin by use of a perfusor. Interventional radiology was contacted with the request for endovascular mechanical thrombectomy, and the patient was transferred to the angiography suite. After bilateral ultrasound-guided access to the common femoral veins, thrombus in the pelvic veins and the inferior vena cava were ruled out using phlebography. The pulmonary trunk was selected using a 5F pigtail catheter, pulmonary artery pressures (PAP) were measured, and digital subtraction angiography (DSA) was performed (Figure 3). PAP measurements revealed elevated pressures with a mean PAP (mPAP) of 42 mm Hg. DSA confirmed CT findings with filling defects and a lack of contrasting of pulmonary parenchyma, especially in the right lower lobe. A stiff guidewire was navigated into the right lower lobe pulmonary artery. The 24F endovascular mechanical thrombectomy device (FlowTriever System) was advanced into the right lower lobar artery. Abundant embolic material was retrieved by repeated aspiration thrombectomy. In the left lung, initial attempts failed. By switching to a FlowTriever catheter containing disks for mechanical thrombus maceration and subsequently continuing aspiration thrombectomy, abundant embolic material was extracted from the left pulmonary arteries as well, particularly from the left lower lobar artery. The thrombi recovered from the pulmonary arteries are shown in Figure 4. Subsequently, a control DSA revealed open and well-perfused pulmonary arteries with only minimal residual thrombotic material (Figure 5). The mPAP immediately decreased by 28 mm Hg from an initial 42 to 14 mm Hg (systolic pulmonary artery pressure (sPAP): 65 → 28 mm Hg; diastolic pulmonary artery pressure: 29 → 6 mm Hg). After the intervention, the patient was hemodynamically stable and free of complaints, only experiencing slight remaining exertional dyspnea. From the patient’s perspective, the intervention was well tolerated. To minimize blood loss during the procedure, an autotransfusion system was used (FlowSaver, Inari Medical, Irvine, CA, USA). Moreover, it was carried out without complications, apart from a small postinterventional venous bleed at the access site in the right groin, which was managed by manual compression. 

**Figure 6:**
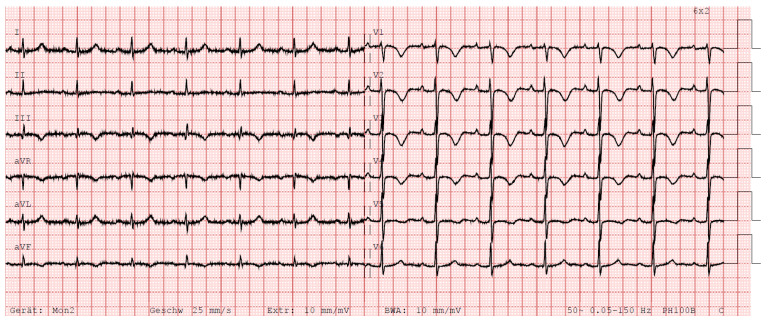
ECG 18 h after intervention. Annotation: sinus rhythm; 82 bpm; SIQIII type; P pulmonale (P amplitude in V2: 0.2 mV); P amplitude in II: 0.12 mV; no right bundle branch block (QRS time: 90 ms); persistent S waves in V5/V6 (S amplitude V6 > 0.3 mV); positive Sokolow–Lyon index for right ventricular hypertrophy (>1.05 mV, here 1.2 mV); R V1, V2 + S I, aVL − S V1 not > 0.6 mV (0.2 mV); R wave peak in V1 > 35 ms (40 ms); T wave inversions in III, aVF, V1–V5; no extrasystoles; ECG, electrocardiogram.

After the intervention, the patient spent a total of 5 days in hospital, including one day in intermediate care and none in the intensive care unit (ICU). Oral anticoagulation with apixaban was started the day after endovascular mechanical thrombectomy (7 days 2 × 10 mg, then 2 × 5 mg). Despite the patient’s symptoms and pulmonary hemodynamics improving significantly, lucid signs of right heart strain could still be found in the ECG approximately 18 h after thrombectomy (Figure 6). 

**Figure 7:**
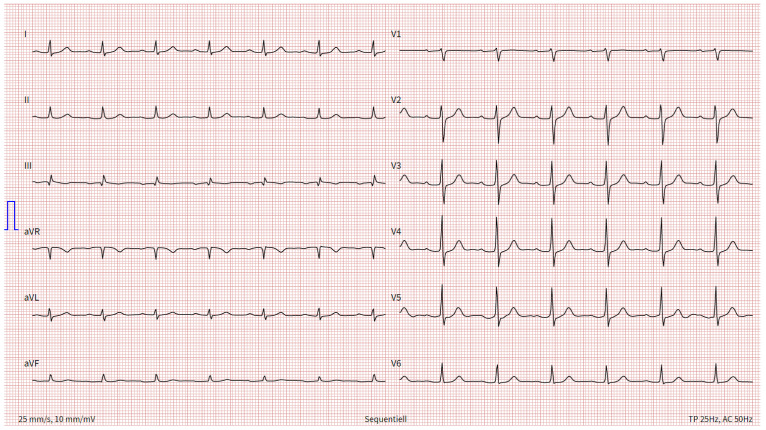
ECG 4 months after intervention. Annotation: sinus rhythm; 78 bpm; left axis deviation; no P pulmonale; P amplitude in II: 0.06 mV; no right bundle branch block (QRS time: 90 ms); small persistent S waves in V5/V6; negative Sokolow–Lyon index for right ventricular hypertrophy (>1.05 mV, here 0.8 mV); R V1, V2 + S I, aVL − S V1 not > 0.6 mV (0.25 mV); R wave peak in V1 not > 35 ms (30 ms); T wave inversion in III; no extrasystoles; ECG, electrocardiogram.

Although the patient’s ECG no longer revealed tachycardia (82 bpm) or RBBB (QRS: 90 ms) or exceeded the threshold of the parameter “R V1, V2 + S I, aVL − S V1” (>0.6 mV, here 0.2 mV), an SIQIII type, persistent S waves in V5 and V6, and a time to peak of R wave in V1 longer than a threshold of 35 ms (40 ms) remained. Furthermore, the S wave in V6 now exceeded a defined threshold for right heart strain (>0.3 mV, here 0.32 mV), TWIs were now additionally seen in leads V4 and V5, and the Sokolow–Lyon index for right ventricular hypertrophy was deemed positive (>1.05 mV, here 1.2 mV). A newly emerged accentuated P wave was also detected in the precordial leads (0.2 mV), by definition a P pulmonale (>0.15 mV in precordial leads) [23]. However, control echocardiography performed later, 4 days after the intervention, no longer showed signs of right heart strain. In addition, the dyspnea disappeared completely by the time of discharge at 5 days after the procedure. In a follow-up visit approximately 4 months after the intervention, the patient presented completely symptom free (NYHA III at diagnosis). His heart rate (129 → 78/min), respiratory rate (20 → 12 breaths/min) and oxygen saturation (94 → 96%) were now unremarkable. Nevertheless, the patient had persistent high blood pressure (149/102 → 154/97 mm Hg) due to his arterial hypertension. Also, high-sensitivity troponin T (43.2 → 13 ng/L), NT-pro-BNP (3538 → 129 ng/L), CRP (13.2 → 7.2 mg/L), creatinine (133 → 110 µmol/L) and eGFR (48 → 60 mL/min/1.73 m^2^) improved. The follow-up ECG was normal. Only a persistent TWI in lead III and small persistent S waves in V5/V6 were still detectable. All other previous ECG changes had resolved (Figure 7). 

Echocardiography also excluded recurrent right heart strain. The values for left ventricular ejection fraction (LVEF, 60%), right atrial pressure (RAP, 5 mm Hg), peak systolic tricuspid regurgitation velocity (TRV, 2.4 m/s), tricuspid annular plane systolic excursion (TAPSE, 18 mm), sPAP (28 mm Hg) and TAPSE/sPAP ratio (0.64) were normal. In combination with the absence of symptoms, chronic thromboembolic pulmonary hypertension (CTEPH), chronic thromboembolic pulmonary disease without pulmonary hypertension (CTEPD without PH) and post-pulmonary embolism impairment (PPEI) could be excluded. In addition, with a value of 6.6, the PEmb-QoL questionnaire demonstrated a high quality of life after PE (best value to be reached—6, worst value to be reached—27) [24]. In a thrombophilia evaluation performed about 4 months after diagnosis, an elevated factor VIII level and hyperhomocysteinemia were detected. In combination with the fulminant initial thromboembolic event, the patient was recommended lifelong anticoagulation with apixaban (dose reduction to 2 × 2.5 mg after 6 months).

## 3. Discussion

### 3.1. Endovascular Mechanical Thrombectomy in Patients with Pulmonary Embolism

An up-to-date registry reports an overall 16% 30-day mortality rate for PE, and only half of the high-risk patients survive [3,25]. This illustrates the demand for immediate and successful PE treatment, which could potentially be achieved by recanalizing therapies [1,14,15]. Currently, the ESC/ERS guideline recommends the use of recanalization therapies only in hemodynamically unstable patients and endovascular mechanical thrombectomy only as an alternative to systemic thrombolysis in case of contraindications or treatment failure [1]. For intermediate–high-risk patients, an ESC consensus statement from 2022 recommends catheter-directed therapy (thrombectomy or local thrombolysis) in the absence of improvement with anticoagulation alone and contraindications to systemic thrombolysis, or in the case of unsuccessful systemic thrombolysis [15]. However, systemic as well as catheter-directed thrombolysis carries a bleeding risk (major bleeding in up to 11.5% and 10% and intracranial bleeding in up to 2.4% and 1.9%, respectively), and many patients have contraindications, resulting in only about one-quarter of high-risk patients receiving thrombolysis [26,27,28,29,30,31,32]. Since endovascular mechanical thrombectomy does not require thrombolytic drugs, the risk of bleeding is reduced significantly [14,16,33]. The optimal treatment approach for intermediate-risk and high-risk PE patients remains unclear. Despite revealing no contraindications to thrombolysis, we found the patient to be a good candidate for endovascular mechanical thrombectomy, since an ESC consensus statement from 2022 recommends catheter-directed therapy (thrombectomy or local thrombolysis) in the absence of improvement with anticoagulation alone in intermediate–high-risk patients [15]. The FlowTriever System is one option for endovascular mechanical thrombectomy, and the first studies using this system revealed promising results in intermediate-risk and high-risk patients (0–11.3% major bleeding, 0% intracranial bleeding, 0–2 days ICU stay and 3–10 days total hospital stay, 47–63% without ICU stay, 0–4.3% 30-day all-cause mortality rate, 3–8 mm Hg average mPAP drop) [34,35,36,37,38,39]. In our patient, mPAP decreased by 28 mm Hg, from an initial 42 to 14 mm Hg, after thrombectomy. No major bleeding occurred. After the intervention, our patient did not stay in the ICU and stayed in hospital for a total of 5 days. Four months after the intervention, he presented as completely symptom free, without electrocardiographic or echocardiographic evidence of recurrent right heart strain or dysfunction and with high quality of life.

### 3.2. ECG in Patients with Pulmonary Embolism

The ECG is one of the first diagnostic tests to be performed in patients with suspected PE, and is able to confirm the suspicion through typical electrocardiographic changes [1,5,6,7]. In a healthy human, the left ventricle masks the right ventricle in an electrocardiogram due to its larger muscle mass. But, as soon as the right ventricle is exposed to increased volume and/or pressure, as it is in PE, the right ventricle pulls the electrical forces more forward, and is able to induce visible electrocardiographic changes. Lead V1 usually shows electrocardiographic pathologies most clearly and promptly, as it is the lead closest to the right heart [40,41,42]. Typical electrocardiographic PE signs are sinus tachycardia or supraventricular tachycardia (e.g., atrial fibrillation), P pulmonale, QRS axis associated with right heart strain (SIQIII or SISIISIII type, right axis deviation > 90°), right bundle branch block (RBBB), qR configuration in V1, TWI, and ST segment depressions or elevations [1,5,6,7]. Unfortunately, ECG parameters are lacking sufficient sensitivity (0.5–77%), specificity (70–100%), positive predictive value (PPV, 23–69%) and negative predictive value (NPV, 64–83%) to definitively diagnose or exclude PE [6,43,44]. Electrocardiographic PE signs sometimes also occur in healthy subjects and are absent in about 15–40% of PE patients [5,6,9,45]. Nevertheless, many studies have pointed out the prognostic value of several ECG parameters in PE patients [5,8,9,11,12,13]. Some of these, which our patient also exhibited, are sinus tachycardia, SIQIII type, RBBB and TWI, which suggest right ventricular dysfunction and are associated with higher mortality and complication rates [11,13,44,46,47]. For example, the more leads that exhibit TWI, the more likely it is for right ventricular dysfunction and death to occur. Kosuge et al. showed that when TWI occurred in seven leads or more, 100% of the patients had right ventricular dysfunction, and 46% died or needed catecholamine support, cardiopulmonary resuscitation or mechanical cardiovascular support. However, it was also found that the more the T waves normalized after therapy, the better the prognosis [47,48,49]. Our patient revealed TWI in seven or more leads (III, aVF, V1–V5) and consecutive right ventricular dysfunction on CTPA, but never needed catecholamine support, cardiopulmonary resuscitation or mechanical cardiovascular support. Previous studies discovered that ECG changes due to PE can vanish after treatment initiation [7,50,51,52], sometimes within 24 h. Alternatively, the ECG can deteriorate initially and improve only after a few days [52,53]. In some cases, the ECG changes persist long-term [52]. Our patient showed five TWIs (III, aVF, V1–V3) at time of diagnosis, seven TWIs (III, aVF, V1–V5) 18 h after intervention, and only one residual TWI (III) at the 4-month follow-up. All other ECG pathologies besides small persistent S waves in V5/V6 had disappeared at this point. We hypothesized that the ECG changes had vanished between the second ECG (18 h after the intervention) and follow-up echocardiography (4 days after the intervention). This fits in well with the fact that the patient’s dyspnea disappeared gradually and only completely after 5 days. Moreover, this demonstrates that the ECG can initially worsen but then improve significantly. Additionally, the present ECG shows that our patient’s prognosis was initially poor, but his prognosis improved significantly after endovascular mechanical thrombectomy.

### 3.3. Role of the ECG in Pulmonary Embolism Sequalae

The ECG has proven useful in the early detection of long-term PE sequelae [54]. Three PE sequelae can be distinguished: CTEPH, CTEPD without PH and PPEI [1,55,56]. Persistent symptoms and/or right ventricular dysfunction from PPEI occur in about every two to six patients, increasing the risk of re-hospitalization, death, and a lower quality of life [55,56,57,58,59,60,61]. CTEPH and CTEPD without PH typically present as unclear exertional dyspnea after PE [1,62,63]. Both are characterized by alterations of the pulmonary vessels but differ in pulmonary hemodynamics (CTEPH: mPAP > 20 mm Hg; CTEPD without PH: mPAP ≤ 20 mm Hg) [62,63]. CTEPH occurs in about 2–3% of PE survivors and is diagnosed with an average delay of 14–15 months [56,64,65,66,67]. An algorithm comprising an assessment of CTEPH probability (risk factors and symptoms), ECG criteria, NT-pro-BNP, and echocardiography was able to diagnose CTEPH with a sensitivity and specificity of 85% and 100%, respectively, (PPV: 100%; NPV: 100%) 4 months post-PE [54]. Therefore, ECG may contribute to the early diagnosis of CTEPH but cannot reliably rule out CTEPH, as the ECG can be completely normal in mild PH [18,40]. In our patient, an interesting ECG parameter (R V1, V2 + S I, aVL − S V1) usually applied to PH patients exceeded a threshold of >0.6 mV at the time of diagnosis, indicating right heart strain. At 18 h and 4 months after the intervention, the threshold was no longer exceeded. In CTEPH patients with chronic right heart strain, this parameter appears able to support a CTEPH diagnosis, indicate therapeutic improvement, estimate pulmonary hemodynamics and predict severe disease with increased mortality [18]. It may also be helpful in PE patients to detect acute (PE) and/or chronic right heart strain (CTEPH).

## 4. Conclusions

To our knowledge, this is the first report on the electrocardiographic course after successful PE treatment with endovascular mechanical thrombectomy using the FlowTriever System. In patients with PE, an ECG can display vivid signs of right heart strain, which can help to suspect PE or assess the patient’s prognosis. The ECG can initially worsen even after successful treatment, but it then improves, as was observed in this case. Moreover, the present case report indicates that endovascular mechanical thrombectomy is highly effective in reducing acute right heart strain as evidenced by a significant and immediate reduction in mPAP, leading to the sustained improvement of right heart function as demonstrated by a largely normalized ECG after about 4 months. However, despite normal echocardiography, freedom from symptoms and good quality of life, two small, but irrelevant, electrocardiographic abnormalities remained after the intervention. An ECG parameter (R V1, V2 + S I, aVL − S V1) that currently is only applied to PH patients with chronic right heart strain may also be helpful in identifying PE patients with acute (PE) and/or chronic right heart strain (CTEPH).

## Data Availability

The data presented in this study are completely contained within the article.
